# A Monocytic Barrier to the Humanization of Immunodeficient Mice

**DOI:** 10.2174/011574888X263597231001164351

**Published:** 2024

**Authors:** Emily J. Du, Marcus O. Muench

**Affiliations:** 1 Vitalant Research Institute, 360 Spear Street, Suite 200, San Francisco, CA, 94105, USA; 2 Department of Laboratory Medicine, University of California, San Francisco, CA, 94143, USA

**Keywords:** Mice, SCID, humanized mice, blood cells, myeloid cells, macrophages, dendritic cells, transplantation

## Abstract

Mice with severe immunodeficiencies have become very important tools for studying foreign cells in an *in vivo* environment. Xenotransplants can be used to model cells from many species, although most often, mice are humanized through the transplantation of human cells or tissues to meet the needs of medical research. The development of immunodeficient mice is reviewed leading up to the current state-of-the-art strains, such as the NOD-*scid*-gamma (NSG) mouse. NSG mice are excellent hosts for human hematopoietic stem cell transplants or immune reconstitution through transfusion of human peripheral blood mononuclear cells. However, barriers to full hematopoietic engraftment still remain; notably, the survival of human cells in the circulation is brief, which limits overall hematological and immune reconstitution. Reports have indicated a critical role for monocytic cells – monocytes, macrophages, and dendritic cells – in the clearance of xenogeneic cells from circulation. Various aspects of the NOD genetic background that affect monocytic cell growth, maturation, and function that are favorable to human cell transplantation are discussed. Important receptors, such as SIRPα, that form a part of the innate immune system and enable the recognition and phagocytosis of foreign cells by monocytic cells are reviewed. The development of humanized mouse models has taken decades of work in creating more immunodeficient mice, genetic modification of these mice to express human genes, and refinement of transplant techniques to optimize engraftment. Future advances may focus on the monocytic cells of the host to find ways for further engraftment and survival of xenogeneic cells.

## 1. INTRODUCTION

Mice humanized through the xenografting of human cells and tissues play an important role in biomedical research, serving as vital resources for investigations on stem cell transplantation [1], new cellular therapies [2–4], as well as for disease modeling of parasitic [5, 6] and virological [7, 8] infections. The construction of humanized mice relies on immunodeficient mouse strains, which, over the past four decades, have undergone major improvements [9–11]. Although immunodeficient mice, such as the widely used NOD-*scid*-gamma (NSG) strain, lack an adaptive immune response, the engraftment of human cells is still limited by immunological barriers and genetic incompatibilities.

Currently, a major barrier to the complete humanization of mice comes from the innate immune system’s ability to recognize xenogeneic cells. There is strong evidence that monocytic phagocytes are capable of rapidly removing human cells from circulation. Although the NOD genetic background imparts a number of advantageous properties to the monocytic cells of many immunodeficient mice, which we further discuss herein, circulating human blood cells are largely unable to avoid being “eaten” by host macrophages. Despite the fact that the circulation of human erythrocytes lasts longer in NSG mice than wild-type strains [12], the majority of red blood cells (RBCs) are cleared from circulation within 24 hours of transfusion. Similar challenges to the survival of xenogeneic cells are likely to be encountered by any cells introduced into the bloodstream.

Macrophage function, however, cannot be knocked out similarly to other cells of the murine immune system, as macrophages are necessary for normal embryonic development. Lack of functional macrophages has been shown to result in either embryonic or gestational fatalities [13, 14]. In this review, we discuss the role of murine monocytic phagocytes as a major barrier to full human hematopoietic reconstitution in mice, including known mechanisms of macrophage-mediated cell rejection and potential methods to improve the survival of circulating human cells.

## 2. A SHORT HISTORY OF THE DEVELOPMENT OF IMMUNODEFICIENT MICE

### 2.1. Early Immunodeficient Mouse Strains with Thymic and Splenic Deficiencies

The development of humanized mice has gone hand-in-hand with the development of mouse strains with more mutations that augment their immunodeficiencies. A spontaneous mutation in the *Foxn1* gene (*Foxn1^nu^*) led to the nude mouse, characterized by a lack of thymic epithelium and defective hair follicles [15, 16]. Athymic nude mice have profound T-cell defects that further result in reduced immunoglobulin production [17]. Subsequently, the Lasat mouse combined the features of the nude mouse with a congenital dominant defect in spleen formation owing to a mutation in the hemimelia gene, *Dh* [18]. Lasat mice have immunoglobulin levels further reduced from those seen in nude mice [17]. Nude and lasat mice were used to model various cancers using established cell lines. However, despite treatment with anti-lymphocyte serum to further reduce immune cells, not all transplanted mice yielded tumors, and no evidence of metastasis was observed [19]. Taking advantage of the immaturity of the neonatal immune system, subcutaneous transplants of leukemic cell lines in neonatal nude mice did observe some localized and disseminated tumor formation, although no engraftment of hematopoietic tissues was observed [20]. Importantly, this study observed slight changes in differentiation markers of the transplanted cells, providing evidence of how growth in culture cannot fully replicate the *in vivo* environment.

### 2.2. Severe Combined Immune Deficiency (SCID) Mice Enable Human Tissue Transplants

Another spontaneous mutation affecting the *scid* gene, discovered in C.B-17 mice, resulted in the development of a line of homozygous C.B-17*scid* mice [21]. The mutation was observed due to serum immunoglobulin measurements that identified four littermates that lacked most immunoglobulin. The hypogamma-globulinemia in these SCID mice was the result of a profound deficiency in T- and B-cells. Some ‘leakiness’ of the immunodeficient phenotype can occur in these mice due to the rare survival of lymphocytes. As the *Prkdc* gene codes for an enzyme used in DNA repair, mice with the *Prkdc*^*scid*^ mutation are also profoundly sensitive to ionizing radiation [22]. SCID mice proved to be better hosts for xenogeneic transplants than nude or lasat mice and could even be engrafted with primary cells and tissues [23–26]. Nonetheless, there remained barriers preventing the robust engraftment of murine bone marrow by human hematopoietic cells. SCID-hu mice, created by the transplantation of human hematopoietic tissues, had mostly localized engraftment of human cells, with few human blood cells detected in the mouse’s circulation.

### 2.3. The Non-obese Diabetic (NOD) Genetic Background Broadens the Immunodeficiency of SCID Mice

Further improvements to the SCID mouse model were achieved by backcrossing C.B-17*scid* mice onto the NOD/ShiLtSz genetic background [27]. These mice could be effectively transplanted *via* intravenous injection, leading to detectable levels of human cell engraftment in the spleen and bone marrow [28, 29]. In part, this was due to decreased NK cell function as well as other immunological deficiencies acquired from the NOD/ShiLtSz genetic background [30–32]. For instance, the NOD/ShiLtSz background imparts a C5 complement deficiency on the NOD-SCID line of mice [33].

### 2.4. Defects in Monocytic Cells in Mice with the NOD Genetic Background

**Fig. (1).**
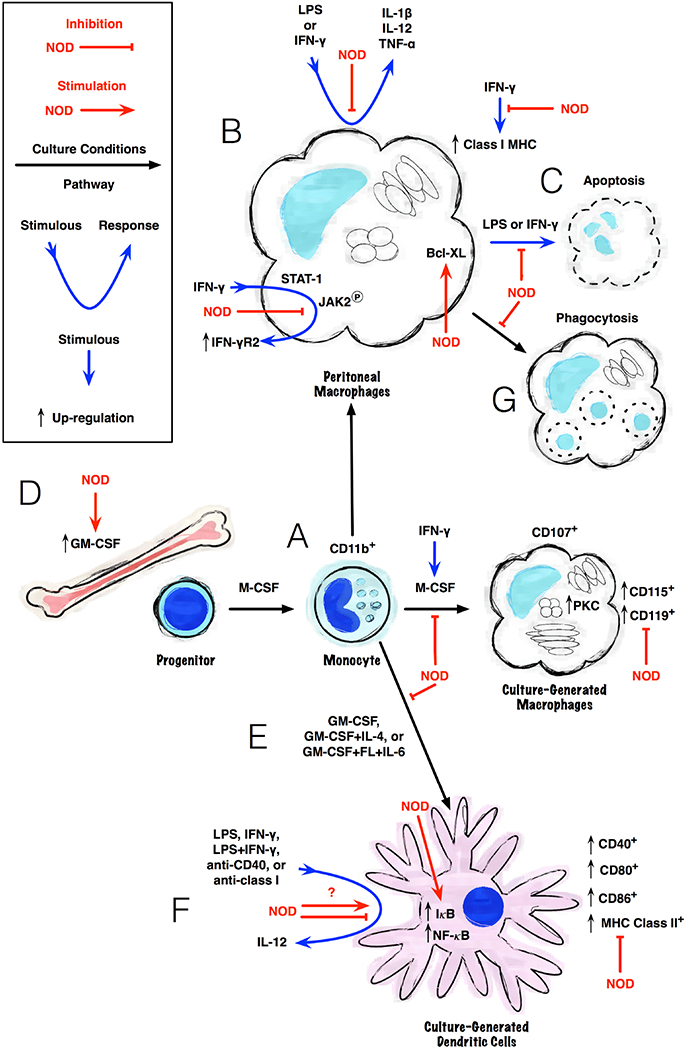
Effects of the NOD/ShiLtSz genetic background on monocytic cells. The growth, maturation, and functional defects of NOD monocytic progenitors, macrophages, and DCs are summarized. Altered cytokine responsiveness diminishes macrophage development *in vitro* (**A**). Stimulated peritoneal macrophages produce less cytokines (**B**), have altered responses to IFN-γ, and are more resistant to apoptosis (**C**). Increased GM-CSF production is observed in the bone marrow of NOD mice (**D**). However, DC development in response to GM-CSF and other cytokines is diminished *in vitro* (**E**). Similar to their macrophage counterparts, stimulated NOD DCs exhibit altered cytokine production and phenotype (**F**). Phagocytosis of apoptotic cells by NOD macrophages was also found to be reduced (**G**). (*A higher resolution / colour version of this figure is available in the electronic copy of the article*).

The NOD/ShiLtSz genetic background also introduced defects in monocytic cell function (Fig. 1). Hematopoietic progenitors in NOD mice have a decreased response to macrophage colony-stimulating factor (M-CSF or CSF-1), which results in diminished growth and maturation of monocytes into macrophages (Fig. 1A) [34]. Bone marrow cells from NOD mice cultured in M-CSF produced fewer lysosome-associated membrane protein 2b (CD107b)^+^ macrophages than from the bone marrow of other mouse strains. However, the more immature monocytic marker, CD11b, was expressed at a similar frequency to other mouse strains, suggesting a defect in macrophage maturation of NOD mice in response to M-CSF stimulation. The culture-generated macrophages also had lower levels of receptors for M-CSF (CD115 or *c-fms*) and interferon (IFN)-γ (CD119), as well as protein kinase C activity resulting from cytokine stimulation [35]. However, the addition of IFN-γ to M-CSF-supported cultures reversed the growth defect, counter to its normal inhibition of macrophage growth, and restored CD107b and cytokine receptor expression in the cultured cells [34, 35].

Reduced levels of TNFα, IL-1β, and IL-12 production were observed from activated peritoneal macrophages of NOD mice (Fig. 1B) [36–38]. NOD macrophages have elevated levels of Bcl-XL and are less likely to undergo apoptosis in response to IFN-γ or lipopolysaccharide (LPS) (Fig. 1C) [38, 39], IFN-γ stimulation of peritoneal macrophages typically increased class I major histocompatibility complex (MHC) antigen expression in mice, but in NOD mice, IFN-γ exposure decreased MHC class I expression [34, 35]. However, MHC class II expression was upregulated by IFN-γ stimulation in a typical manner. Signal transducer/activator of transcription (STAT)1 intracellular signaling was observed to be diminished in response to IFN-γ but not in response to IFN-α/β, resulting in low JAK2 phosphorylation and poor upregulation of IFN-γ receptor 2 (IFNGR2) [38].

Additionally, an increase in granulocyte-macrophage colony-stimulating factor (GM-CSF) expression has been noted in the bone marrow of NOD mice (Fig. 1D) [40]. GM-CSF is a pleiotropic cytokine that acts as a myelopoietic growth factor of hematopoietic stem cells throughout monocyte development [41–43]. GM-CSF also affects the growth, differentiation, and function of mature tissue macrophages [44–47].

GM-CSF further supports the early stages of monocyte differentiation into dendritic cells (DCs) [48, 49]. DCs are a monocytic subset that are specialized in antigen-presentation [50]. Lee *et al.* observed a defect in the capacity of NOD bone marrow cells to develop into DCs when cultured with GM-CSF (Fig. 1E) [51], reminiscent of the defects in macrophage development. In contrast, a proclivity to generate Gr-1^+^CD11b^+^ neutrophils from NOD bone marrow cultures has also been observed [52]. The DCs generated from NOD bone marrow had reduced levels of CD40, CD80, CD86, and MHC class II on their surfaces, which impacted their ability to present antigens and provide co-stimulation to T-cells [51]. However, Boudaly *el al.* still observed defective DC maturation in bone marrow cultures supported by GM-CSF+IL-4 or spleen cell cultures supported by GM-CS-F+flk-2/flt3 ligand (FL)+IL-6 [53].

DCs generated from NOD progenitors in culture have also been reported to produce less IL-12 when stimulated with either LPS, IFN-γ, LPS+IFN-γ, anti-CD40 antibody, or anti-class I MHC antibody (Fig. 1F) [51, 54]. These defects in DCs could be partially reversed by further addition of IL-4 to the cultures [51]. However, Weaver *et al.* observed increased IL-12 secretion and hyperactivity of IκB and NF-κB in culture-generated DCs from NOD mice [55]. These signaling factors are known regulators of IL-12 transcription. Others have reported similar increases in IL-12 production [53, 56, 57]. It is possible that differences in culture conditions and duration account for the conflicting results. There is an inherent complexity to cytokine and immune cell signaling in mixed cell cultures.

Defects in the phagocytic function of NOD macrophages and DCs may be more relevant to xenogeneic cell transplantation in immunodeficient mice than the growth and maturational defects of these cells. Lee *et al.* reported that the endocytic activity of NOD DCs was preserved [51]. This was measured from the ability of culture-generated cells to engulf labeled-dextran. However, the ability of NOD macrophages to engulf apoptotic cells was shown to be reduced, slightly more so for female than male mice (Fig. 1G) [58]. Opsonization of apoptotic thymocytes with anti-CD3 antibody did have a slight enhancing effect on phagocytosis but did not increase to levels comparable with Balb/c mice. Interestingly, the ability of NOD macrophages to phagocytose polystyrene beads was not impaired. As both the annexin V and Fc-receptor-mediated pathways are attenuated in NOD mice, the defect in NOD cells may be a common component for both pathways or another consequence of the maturational defects of NOD monocytic cells.

### 2.5. NSG Mice have a Complete Lack of Lymphocytes and an NOD Genetic Background

NOD-SCID mice, despite being more immunodeficient than SCID mice, still have residual NK-cell activity and a low level of lymphocyte leakiness. The next notable advance in immunodeficient mice came with the introduction of X-linked severe combined immunodeficiency resulting from a null allele of the IL-2 receptor common γ-chain (IL2Rg, CD132) [59, 60]. These NSG (NOD.Cg-*Prkdc^scid^ Il2rg*^*tm1Wjl*^/SzJ) mice lack all lymphocytes due to a complete lack of cytokine-mediated signaling by receptors that use the CD132 receptor sub-unit. These cytokines include IL-2, IL-4, IL-7, IL-9, IL-15, and IL-21 [59]. Transplantation of human hematopoietic stem cells, obtained from umbilical cord blood, into NSG mice, resulted in high levels of human chimerism in the bone marrow, spleen, and among nucleated peripheral blood cells [59]. Multi-lineage engraftment was also achieved using human CD34^+^ mobilized peripheral blood cells, with engraftment levels notably higher than in NOD-SCID mice [60].

## 3. AVAILABLE STRAINS OF IMMUNODEFICIENT MICE

### 3.1. NSG and other Similar Mouse Strains

Currently, NSG mice represent the most widely used immunodeficient mouse strain. Literature searches for the term “NSG mice” retrieve more than three times the amount of published articles than other comparable strains [62]. Other phenotypically similar strains that are available include Charles River’s NCG (NOD-*Prkdc*^*em26Cd52*^ *Il2rg*^*em26Cd22*^/NjuCrl) mouse, created by CRISPR editing of the *Prkdc* and *Il2rg* genes in the NOD/Nju mouse, as well as the Central Institute for Experimental Animals’ (CIEA) NOG (NOD.Cg-*Prkdc*^*scid*^ *Il2rg*^*tm1Sug*^/ShiJic) mouse (Table 1). NOG and NSG mice differ in their *Il2rg* mutations, as NOG mice possess an *Il2rg* gene expressing a protein that can bind to cytokines but cannot signal, while NSG mice have a complete null mutation, where no Il2rg is expressed [63].

Mice with knockouts of recombination activating genes 1 and 2, or *Rag1* and *Rag2*, have been developed that are phenotypically similar to *scid*-based immunodeficient mouse strains. *Rag2* expresses a protein required for generating functional antigen receptors in lymphocytes; *Rag2* knockouts subsequently prevent the maturation of lymphocytes, resulting in a similar phenotype to *Prkdc-scid* [62]. *Rag2* knockout-based strains are non-leaky and possess a higher tolerance for ionizing radiation [63]. Jackson Laboratory’s BRG mouse and Taconic’s DKO combine the *Rag2* knockout with *Il2rg* deletion, resulting in mice that lack B, T, and NK cells [64, 66].

### 3.2. The Genetic Humanization of Immunodeficient Mice

In addition to efforts to create mouse strains with ever greater immunodeficiency, immunodeficient mice have been genetically humanized in order to provide support for human hematopoiesis and mature blood cells. NSG-3GMS (NOD.Cg-*Prkdc*^*scid*^ *Il2rg*^*tm1Wjl*^ Tg(CMV-IL3, CSF2, KITL-G)1Eav/MloySzJ) mice express the human cytokine transgenes IL-3, GM-CSF, and stem cell factor (SCF or KL), which support myeloid development [67]. These human cytokines were chosen because they do not affect the growth of mouse cells. Furthermore, kit-ligand (KL) transgenic mice have been generated on the NSG background (hKL-T-g-NSG (NOD.Cg-*Prkdc*^*scid*^ *Il2rg*^*tm1Wjl*^ Tg(PGK1-KITL-G*220)441Daw/SzJ)); mice of this strain have been shown to possess enhanced human HSC engraftment [68]. Additional mouse substrains on NSG and similar backgrounds expressing human cytokine genes have also been developed.

hSIRPa-DKO (C;129S4-*Rag2*^*tm1.1Flv*^ *Il2rg*^*tm1.1Flv*^Tg (SIR-PA)1Flv/J) mice express human signal-regulatory protein alpha (SIRPα) on a *Rag2* null and *Il2rg* null background [69], which are discussed in further detail below. Additionally, MISTRG (C;129S4-*Rag2*^*tm1.1Flv*^ *Csf1*^*tm1(CSF1)Flv*^ *Csf2/Il3*^*tm1.1 (CSF2,IL3) Flv*^ *Thpo^tm1.1 (TPO)Flv^ Il2rg^tm1.1 Flv Tg(SIRPA)^*1Flv/J) mice have been developed, which express human knock-ins of M-CSF, IL-3, and thrombopoietin (TPO), and a bacterial artificial chromosome transgene for human SIRPα [70].

## 4. THE LIMITS OF HUMANIZATION OF THE HEMATOPOIETIC SYSTEM

### 4.1. Hematopoietic Chimeras Reveal the Blood to be the Least Permissive Tissue for Engraftment

**Fig. (2).**
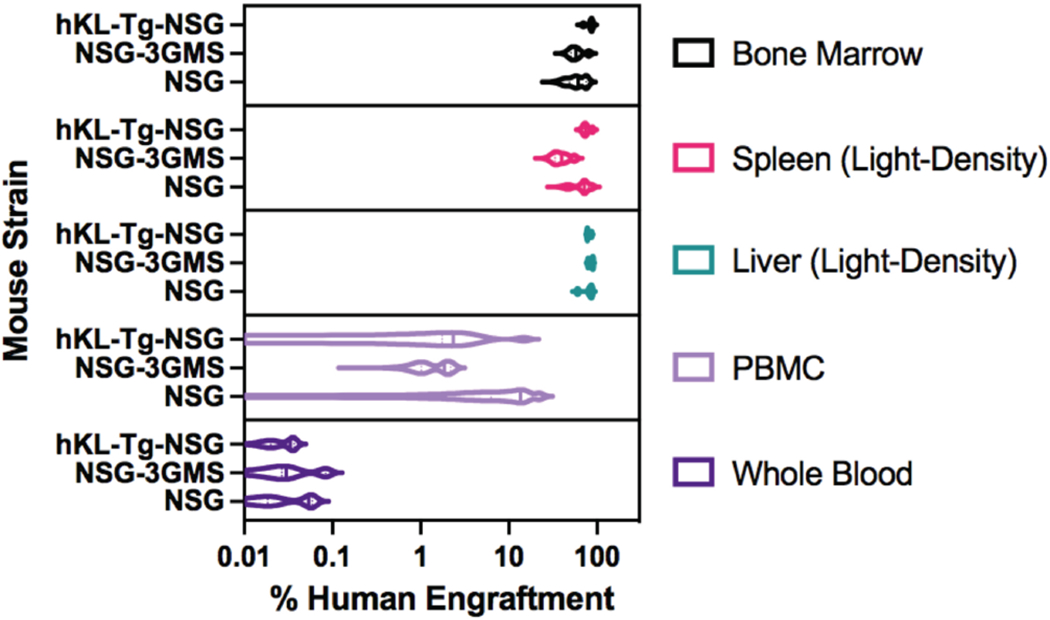
Human hematopoietic chimerism in three strains of immunodeficient mice after stem cell transplant. Mice were transplanted with human fetal bone marrow and analyzed 12 weeks later. Violin plots show the frequency of human β2-microglobulin^+^ cells among whole bone marrow, light-density splenocytes, light-density liver cells, light density blood (PBMC), and whole blood. NSG mice were compared as hosts to two mouse strains expressing human-specific cytokines. Violin plots compare engraftment among the three mouse strains for each tissue (n=5). Note % engraftment is on a log scale. These data were previously published in [71]. (*A higher resolution / colour version of this figure is available in the electronic copy of the article*).

NSG mice allow extensive humanization of the hematopoietic system [59, 60] when transplanted with fetal or neonatal stem cells. Despite abundant human chimerism in the bone marrow and high levels of leukocyte engraftment in the spleen and liver, circulating blood cells remain primarily of mouse origin. This is evident in a comparison performed by our laboratory of human chimerism levels in different tissues after human fetal bone marrow transplantation, which clearly showed the discrepancy between the extensive hematopoietic engraftment in the bone marrow and the predominantly murine composition of the blood (Fig. 2) [71]. The comparison was made in three strains of NSG mice: standard NSG mice, as well as two strains expressing human cytokines. Although the frequency of human cells was over 80% in the bone marrow of hKL-Tg-NSG mice and nearly as high among the mononuclear cell fraction of the liver and spleen, the frequency of peripheral blood mononuclear cells (PBMCs) in these mice was <5%. This is remarkable when one considers that the immuno-deficiency of these mice should provide a very permissive environment for long-lived human lymphocytes to survive. However, species-specific factors affecting the survival and growth of human lymphocytes are undoubtedly at play [72]. When red cells were included in the analysis, chimerism levels among whole blood cells were <0.1%. In the bone marrow, even in hKL-Tg-NSG mice (which express KL, a critical erythropoietic cytokine), erythropoiesis was only weakly supported [71], which may partially account for the low numbers of human erythrocytes in circulation.

### 4.2. Rapid Clearance of Transfused Human Blood Cells

**Fig. (3).**
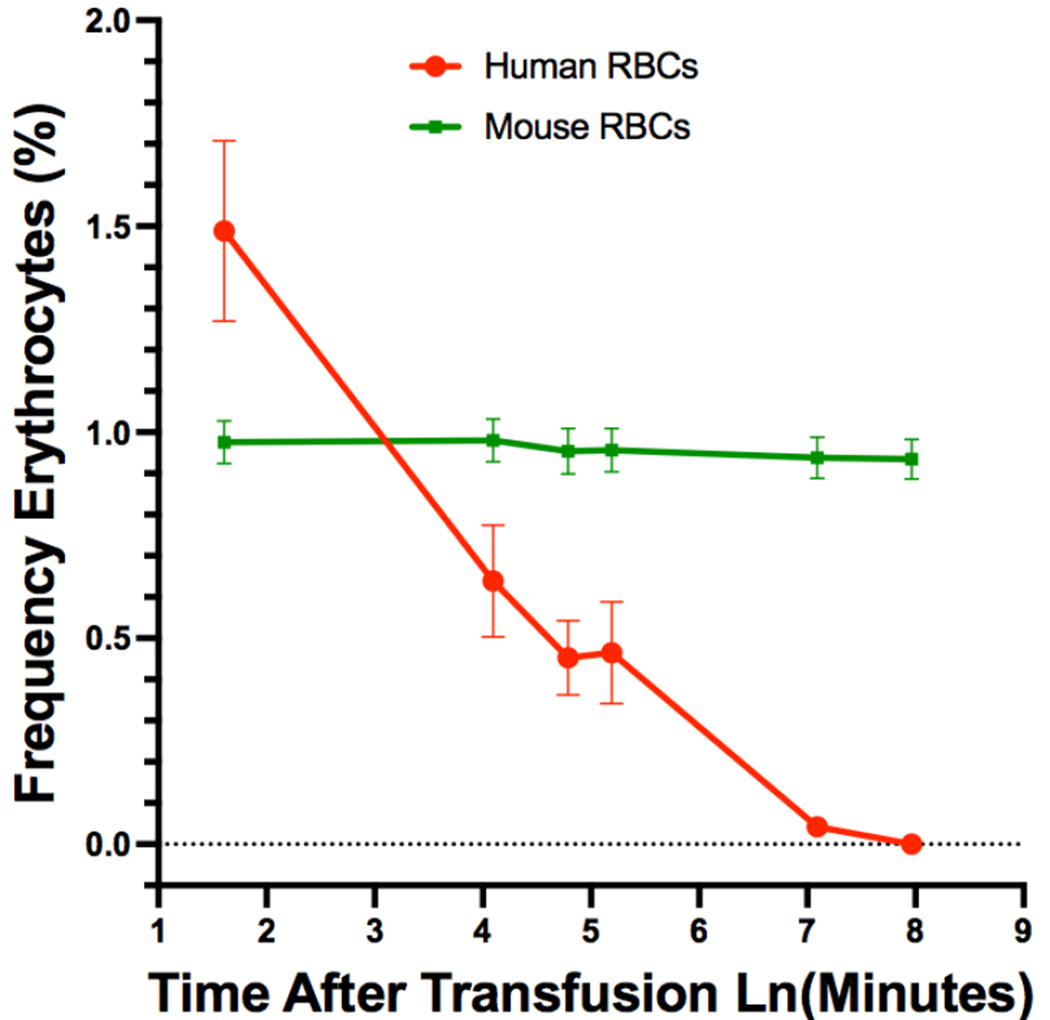
Rapid clearance of human erythrocytes from the circulation of NSG mice. Mice were transfused with a mixture of human and mouse red cells. Donor mouse erythrocytes were obtained from mice that expressed enhanced green fluorescence protein so that donor cells could be distinguished from the host’s own red cells. The starting ratio of human:mouse erythrocytes transfused was >4:1, which was already greatly reduced by the first measured time point after transfusion (5 minutes). The frequencies of mouse erythrocytes remained relatively stable over the course of the experiment compared to the rapid decline in circulating human erythrocytes. Note that a natural log transformation of the measurement times is used to better visualize early measurements (5, 60, 120, and 180 minutes) while also allowing for the display of the two last time points (20 and 48 hours). These data were previously published in [12]. (*A higher resolution / colour version of this figure is available in the electronic copy of the article*).

Direct evidence for rapid clearance of human cells from the circulation of immunodeficient mice was shown by analyzing the clearance of transfused xenogeneic blood cells [12, 73, 74]. Most human leukocytes, erythrocytes, and platelets transfused into NSG mice disappeared within a day [12]. Indeed, a notable loss of human red cells was observed within minutes of transfusion (Fig. 3). The human erythrocytes were found to rapidly accumulate in the lung and liver, but not so much in the spleen [12], which was interesting given the abundance of macrophages in the spleen and the role of this organ in the removal of old erythrocytes (for review, see [75]). These observations underscore the physiological barriers that still exist and prevent performing anything other than short-term studies on human blood products for transfusion. Moreover, realizing the goal of fully humanizing the hematopoietic and immune systems of mice through stem cell transplantation requires the survival of human cells in circulation. It should be noted, however, that despite the rapid clearance of human blood cells from the circulation, the persistence of human leukocytes in tissues after transfusion allows for sufficient engraftment of these cells to model facets of the human immune system [24, 76–79]. Other cell and tissue types are likely to be affected by the same mechanisms that limit hematopoietic engraftment, especially for transplants performed *via* an intravascular route.

## 5. MONOCYTIC CELLS ARE AN INNATE IMMUNE BARRIER TO XENOTRANSPLANTATION

### 5.1. Leukocytes can Engraft and Proliferate in Immunodeficient Mice Despite Rapid Clearance

The barriers to a more complete humanized mouse are inherently multifactorial. NSG and phenotypically similar strains retain an intact, albeit altered, myeloid-derived innate immune system. Phagocytosis by these myeloid cells is the likely means by which most xenogeneic cells are removed from immunodeficient mice, as most other immune pathways that could plausibly eliminate foreign cells do not exist. The process of cell elimination appears to be most efficient for circulating xenogeneic cells based on the examples discussed above, demonstrating high levels of human hematopoietic chimerism in the bone marrow yet low levels of circulating human blood cells [71].

NOD-background strains, such as NSG and NOG, can support limited engraftment of human PBMCs [78, 79] despite the rapid disappearance of circulating leukocytes after transfusion [12]. There are a number of important observations regarding these PBMC-engrafted mice. First, these mice are primarily engrafted by T-cells and, to a lesser extent, B-cells [80]. Lymphocytes are long-lived and are able to replicate, suggesting that they may be the lucky descendants of a small number of cells that survived to engraft and proliferate. Most of the T-cells have the phenotypic profile of activated T-cells, further suggesting stimulation and proliferation [78]. Indeed, PBMC-engrafted mice may be best viewed as a model of xenogeneic graft-versus-host disease, although they do offer opportunities for other lines of research. It is also worth noting that although we observed a rapid decline in leukocytes after transfusion, we also observed fluctuations in circulating leukocyte numbers in some animals during the hours immediately after administration [12]. We referred to this phenomenon as the rebound effect and speculate that it is the result of the sequestration of leukocytes in tissues, perhaps by adherence to the endothelium. Thus, although enough transplanted hematopoietic stem cells or transfused leukocytes can survive to engraft a mouse, many, if not most, of the injected cells are likely to perish within the first day.

The stark difference in fates between intravenous cell transplants and tissue xenografts was evident in the first successful hematopoietic xenograft models established in SCID mice. In order to achieve PBMC engraftment in SCID mice, high numbers of cells were delivered intraperitoneally rather than intravenously [81, 82]. Construction of SCID-hu mice by co-transplantation of human fetal thymus and liver cells/fragments under the kidney capsule results in significant growth of human thymic tissue at the site of transplantation [23]. Although circulating T-cells are detected in such mice, they eventually disappear as the graft diminishes. SCID mice implanted with human fetal bone fragments also have the majority of their human cells localized to the graft, with few cells detected in the blood [25]. Even though tissue grafts are relatively more resistant to rejection by mouse cells, human tissue grafts, in particular, bone grafts, do contain mouse cells. The decline of the grafts is correlated with increased numbers of mouse leukocytes. Moreover, in xenogeneic transplant models in which the hosts were not immunodeficient, macrophages were found to be the dominant host cell type observed during graft rejection [83–85]. Conversely, one can also speculate that monocytic cells contained within human grafts may also play a role in protecting the human tissues from host cell rejection.

It is worth mentioning that many studies examining chimerism levels in the blood focus on PBMCs, with data often reported as the percentage of human cells among PBMCs. Although this is a straightforward and reasonable method of data presentation, it must be kept in mind that due to the absence of any lymphocytes, the dominant PBMC populations in immunodeficient mice are monocytes. Monocytes usually represent only 10-30% of PBMCs when lymphocytes are present. Thus, if a human cell transplant were to reconstitute all the missing leukocyte elements, human cells should outnumber mouse cells at least several fold to approach what may be considered normal blood count values.

### 5.2. Macrophage Depletion Increases the Survival of Xenogeneic Leukocytes

The role of macrophages in the clearance of xenogeneic cells was demonstrated in mice treated intravenously with liposome-encapsulated dichloro-methylene diphosphonate, also referred to as clodronate, which could temporarily eliminate macrophages within 24 hours of administration [86–88]. Liposomes are cleared from the circulation by macrophages through endocytosis [89]. Within the macrophage, endosomes fuse with lysosomes; it is within these lysosomes that the liposomes are broken down and release their toxic payload. The clodronate is then hydrolyzed to an ATP analog that cannot be further hydrolyzed by the cell, leading to cell death by apoptosis or necrosis [90].

Analysis of macrophage populations in the spleen of mice treated with clodronate liposomes revealed the broad sensitivity of various macrophage populations to depletion but different kinetics in recovery [91]. Red pulp macrophage numbers began to recover around day 6 after treatment and reached normal levels around day 10. White pulp macrophages, some of which resisted the liposome treatment, also recovered in a similar time frame (day 8). However, marginal zone macrophage numbers took about a month to recover with lingering functional defects. Reconstitution of splenic macrophage populations is through both recruitment and proliferation of surviving macrophages [92]. Accordingly, clodronate liposome treatment is an effective means to rapidly deplete macrophage populations, with their recoveries beginning about a week after treatment.

Fraser *et al.* used clodronate liposomes to examine that macrophage depletion could enhance engraftment of PBMCs in SCID mice, providing the first evidence of the importance of macrophages in xenogeneic cell rejection in immunodeficient mice [93]. They further demonstrated that in SCID-hu mice with thymic constructs, clodronate liposome treatment could temporarily enhance the survival of circulating human T-cells as well as marginally increase these cells in the spleen. Terpstra *et al.* extended these observations, showing that engraftment of acute myeloid leukemia cells, as well as hematopoietic stem cells from umbilical cord blood, was enhanced by macrophage depletion [94]. They performed their work using SCID mice, which were also pretreated with total body irradiation with or without clodronate liposome treatment. Similar results were observed by Verstegen *et al.*, who showed that stem cell transplantation in SCID mice preconditioned with irradiation and depletion of macrophages had human chimerism levels similar to NOD-SCID mice treated only with whole-body irradiation [95, 96]. Macrophage depletion was also found effective in enhancing PBMC engraftment in Rag2^−/−^ γc^−/−^ mice [94]. Additionally, the depletion of macrophages was effective in increasing the survival of human cells in NSG mice despite their already compromised monocytic cells. Both RBC and platelet reconstitution were enhanced by macrophage depletion [97, 98]. In total, these data support the idea that macrophages are one of the main impediments to xenogeneic cell survival in mice lacking lymphocytes and other immunodeficient genetic profiles.

### 5.3. Other Phagocytic Cell Types may also be Involved in the Clearance of Xenogeneic Cells

Our discussion of phagocytic cells has concentrated on monocytic cells - monocytes and macrophages. Additional types of “professional” phagocytic cells exist, including neutrophils and DCs [99], that are not depleted by clodronate liposomes [91, 100]. Neutrophils, DCs, as well as “amateur” phagocytes, such as endothelial cells, have been implicated in the clearance of human cells [101, 102]. It is also worth noting that mouse neutrophil and monocyte numbers were observed to increase in circulation about 8 hours after human RBC transfusion [12]. Evidence has been presented for a secondary role for neutrophils and possibly endothelial cells in the phagocytosis of human RBCs, but only when the primary threat posed by macrophages has been removed by chlodronate liposome treatment [103].

It is noteworthy that a recent study by Culemann *el al.* showed that clodronate liposomes also affect neutrophils [104]. Neutrophils were found to ingest clodronate liposomes, which affected their ability to perform phagocytosis as well as other functions, such as modulation of cell surface receptors, migration, cytokine production, production of reactive oxygen species, and generation of neutrophil extracellular traps. These profound effects were summarized as a ‘stunning’ effect on neutrophils, resulting in an anti-inflammatory effect in addition to the well-known depletion effect on monocytic cells.

### 5.4. Monocytic Cells Represent a Spectrum of Functionally Diverse Cells that are Critical for Survival

Macrophages are among the first blood cell lineages to be formed in mice, with macrophage progenitors appearing simultaneously alongside primitive erythroid progenitors [105]. Tissue macrophage populations are now understood to possess a dual origin: 1. Embryonic macrophages originating from myeloid progenitors that seed tissues during development, effectively bypassing the monocyte stage, and 2. Monocytes in circulation enter tissues and differentiate into tissue macrophages. Embryonic-derived tissue macrophage populations are capable of repopulating with minimal replenishment from circulating monocytes [106–108]. Such lineages in mice include Kupffer cells [109], alveolar macrophages [110, 111], and, to some extent, splenic red pulp macrophages [112]. However, the functional differences between embryonic lineage-derived tissue macrophages and circulating monocyte-derived tissue macrophages are currently minimally defined.

Both embryonic-derived macrophages and macrophages derived from circulating monocytes can be ablated with methods, such as the administration of clodronate liposomes [113, 114] or using genetically modified mice containing the Macrophage Fas-induced Apoptosis (MaFIA) gene construct [115]. Treatment of mice with a small molecule dimerizer is used to selectively deplete cells expressing the MaFIA transgene, namely monocytic cells. Tissue-resident macrophage populations depleted using these and similar methods are able to be replenished by circulating monocytes, similar to how inflammation recruits circulating monocytes to repopulate tissues, such as the spleen and liver [116].

Macrophages are characterized using Mills’ classical M1 and alternative M2 paradigm [117]. M1 macrophages are characterized by pro-inflammatory markers, while M2 macrophages are anti-inflammatory and participate in tissue repair pathways [117, 118]. Additionally, M2 macrophages are further classified into subtypes M2a-d based on responsive differences and variation in marker expression [119, 120]. However, as macrophages have been shown to adopt intermediary phenotypes [121–123], the classification system proposed by Mills *et al.* has been adapted to encompass a continuum of macrophage function, with the M1 and M2 phenotypes serving as poles on the ends of a spectrum. As macrophage phenotypes are also affected by tissue environment, and tissues often contain heterogeneous macrophage populations, the M1/M2 continuum falls short in its ability to accurately represent macrophages’ complexity and diversity.

A defining element of the monocytic barrier is the inability to knock out murine macrophages similar to other cell lineages of the murine immune system. Macrophages have many functions beyond immune surveillance and are essential for embryonic development [124, 125]; functional macrophage knockouts in mice result in embryonic lethality [13, 14]. Among their many roles, macrophages support hematopoiesis, regulating the bone marrow hematopoietic stem cell niche, especially during inflammatory stress [126]. Central macrophages are also requisite players for the formation of erythroblastic islands during erythropoiesis; each central macrophage is surrounded by erythroblasts and facilitates erythroblast maturation into enucleated erythrocytes [75, 127, 128]. Given the multitude of critical functions performed by monocytic cells, efforts to deplete or eliminate these cells in immunodeficient mice can have both beneficial and negative effects on human chimerism.

## 6. MOLECULAR MECHANISM EMPLOYED IN THE CLEARANCE OF XENOGENEIC CELLS BY MONOCYTE CELLS

**Fig. (4).**
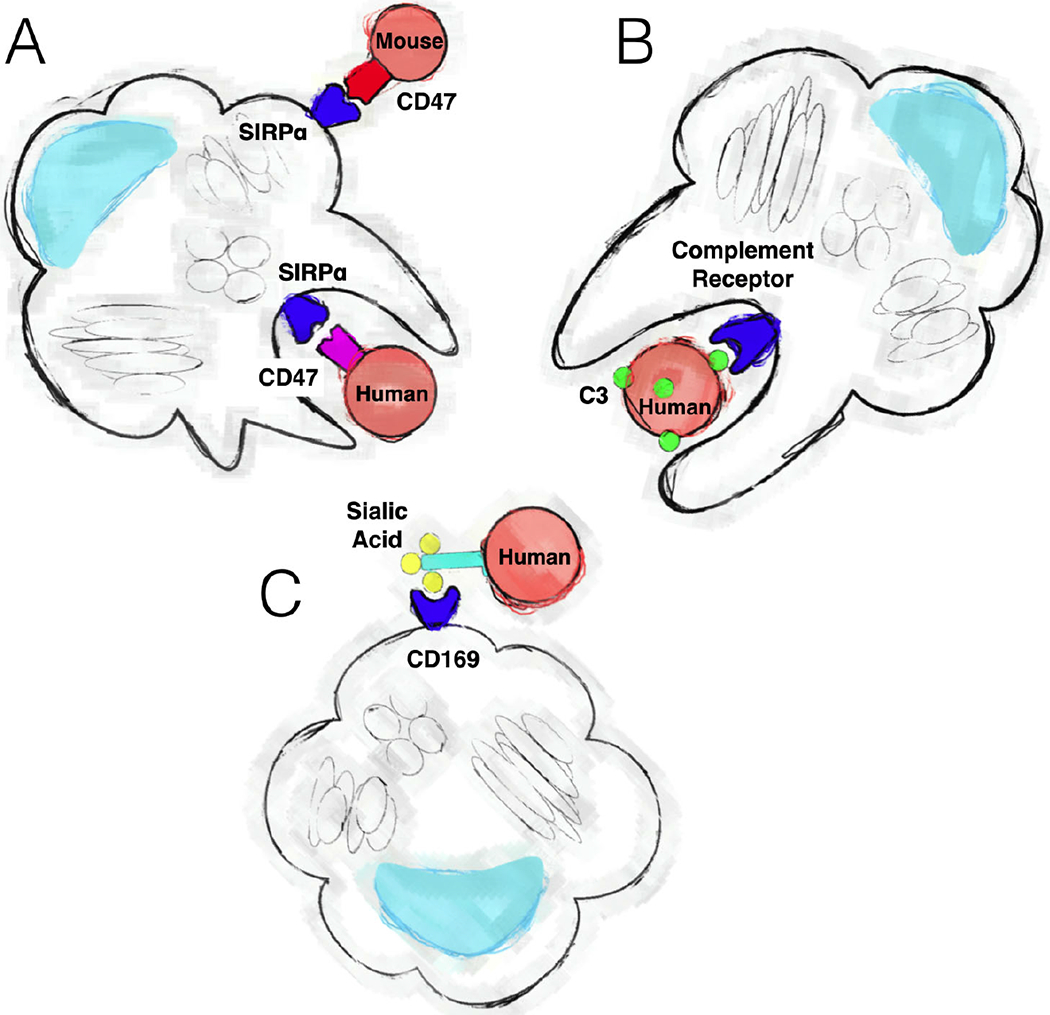
Mechanisms by which mouse monocytic cells identify human cells for removal. Mouse macrophages receive inhibitory signals from CD47 expressed on most cells (**A**). Human CD47 does not provide as strong of a signal through SIRPα receptor on macrophages as mouse CD47, which may result in phagocytosis. Opsonization of human cells with complement 3 components (C3) can also lead to phagocytosis by macrophages (**B**). Mouse macrophages may also bind human cells based on carbohydrate moieties, such as sialic acid binding by CD169, which may foster phagocytosis of the human cells (**C**). (*A higher resolution / colour version of this figure is available in the electronic copy of the article*).

There are a few known or possible mechanisms by which clearance of xenogeneic cells occurs by murine macrophages, as discussed below (Fig. 4).

### 6.1. CD47 and Signal Regulatory Protein-α (SIRPα)

CD47 is a key regulator of self-identification. CD47 is widely expressed, especially in cells of the myeloid lineage (Fig. 4A). Interactions between CD47 and SIRPα (CD172α) play an important role in modulating the phagocytosis of human cells in mice [129–131]. SIRPα is also widely expressed on hematopoietic cells, including monocytes, macrophages, DCs, granulocytes, mast cells, and subsets of B-cell and myeloid progenitors, including stem cells [132–134]. The CD47-SIRPα interaction is widely accepted as a “do not eat me” signal, preventing macrophages expressing SIRPα from engulfing CD47-expressing cells. Incompatibility between this ligand-receptor pair as a result of xenotransplantation can cause graft rejection [135].

There are different alleles for the murine *Sirpa* gene; mice with an NOD genetic background express a polymorphism of *Sirpa* whose protein binds more strongly to human CD47 than the allele found in C57BL/6 or NOR mice [131]. Stronger binding results in greater inhibition of cell clearance in mice sharing the NOD genetic background compared to mice expressing the alternative allele. Furthermore, the NOD *Sirpa* allele was tested in immunodeficient mice on the C57BL/6 background, which lacked the assortment of immunodeficiencies associated with NOD-background mice [136]. These mice, termed BRGS, showed enhanced engraftment of human stem cells, demonstrating the importance of the NOD *Sirpa* allele to the xenograft permissive phenotype of immunodeficient mice sharing the NOD genetic background.

In order to confer cross-reactivity onto non-NOD background mice, mice with humanized *SIRPα* have also been developed. Strowig *et al.* constructed hSIRPa-transgenic double-knockout (hSIRPa-DKO) mice using bacterial artificial chromosomes, with the double knockout moniker reflecting deficiencies in the *Rag2* and *Il2rg* genes. Transplantation of newborn, irradiated hSIRPa-DKO mice with CD34^+^ human fetal liver cells resulted in similar levels of engraftment to NOD-background NSG mice [69].

Our group tested the hSIRPa-DKO strain with human hematopoietic stem cell transplants derived from human fetal bone marrow [71]. For reasons that were not entirely clear, hematopoietic engraftment in these mice was noticeably worse than with NSG mice. One possibility is that other genetic elements of the NOD genetic background are, indeed, important for achieving high levels of human engraftment. However, this is in contrast with the findings of Yamauchi *et al.*, showing that the nature of the *Sirpa* allele is of preeminent importance over other NOD traits [136]. It was also noted that these mice became readily obese and were poor breeders. Whether obesity contributed to poor engraftment was not determined.

### 6.2. Opsonization by Complement

Complement regulatory proteins can play a role in the rejection of human cells from mouse circulation. The complement system is a highly conserved component of innate immunity consisting of multiple protein cascades that modulate the inflammatory response, tag pathogens for phagocytosis, and attack pathogen membranes [137]. Although many immunodeficient mouse models, such as the widely used NSG strain and other strains on the NOD background, lack hemolytic complement protein C5 [33], which is necessary for the formation of the membrane attack complex, other proteins in the complement cascade pathways upstream of C5 remain functional. Most importantly, C3, which functions as a critical signal amplifier for complement activity, is a potential modulator of xenogeneic rejection between humans and the current generation of immunodeficient mice (Fig. 4B).

C3 deposition on hRBCs was initially observed by Isihara *et al.* in SCID mice; interestingly, this finding was particular to human erythrocytes, as C3 deposition was not observed on bovine or equine RBCs [138]. Chen *et al.* demonstrated the effect of complement depletion with cobra venom factor on the survival of transfused hRBC in NOD/SCID mice. Cobra venom factor, a structural analogue of C3, forms stable C3 and C5 convertase with human factor B, activating and depleting C3 and C5 [139]. Complement depletion by cobra venom factor did not significantly improve hRBC survival on its own but successfully prolonged the survival of hRBCs in circulation when combined with macrophage depletion *via* clodronate liposomes [103].

Furthermore, Yamaguchi *et al.* described a substrain of NOG mice with a truncated C3 gene (NOG-C3^ΔMG2-3^) showing prolonged survival of transfused hRBCs and improved hRBC reconstitution from engrafted human hematopoietic stem cells. In this study, gadolinium chloride (GdCl_3_) was used as the macrophage depletion mechanism rather than clodronate liposomes due to GdCl_3_’s lower toxicity [140]. Both Chen *et al.* [103] and Yamaguchi *et al.* observed a synergistic effect between mouse macrophage depletion and complement depletion. The mechanisms of complement-mediated clearance, thus, also appear to involve C3-enhanced phagocytosis by cells other than macrophages.

Additionally, membrane-bound complement regulatory proteins, such as CD46 and CD55, may be molecules of further interest in the context of xenotransplantation. CD55, or decay accelerating factor (DAF), negatively regulates complement activation by associating with C3b to prevent the formation of C3 convertase (C3bBb), interfering with C3’s positive feedback loop and protecting the cell from complement activation [141], Cross-species reactivity has been identified between human CD55 and murine complement proteins [142, 143]. Harris *et al.* showed that inhibition of human CD55 enhances lysis by complement *in vitro*.

On the other hand, CD46, or membrane cofactor of proteolysis (MCP), modulates the cleavage of C3b to inactivated C3b (iC3b) by acting as a cofactor for the protease factor I [144–146]. CD46 is expressed ubiquitously in human cells but is isolated to testicular germ cells in mice [144]. Importantly, mice express the rodent-specific membrane protein Crry (CR1-related y), a regulator of complement activation that appears to serve as a functional analog of both human CD46 and CD55 [147, 148]. Crry is not a genetic homolog of human CD46 or CD55 and displays a divergence in structure but convergence in function to its human convertase formation, preventing counterparts [149, 150]. The nuanced differences between human and murine complement regulatory systems are areas of further investigation for potential molecular mechanisms of complement-mediated xenogeneic cell rejection.

### 6.3. Sialic-acid Residues

Other possible mechanisms for rapid xenogeneic cell clearance, in particular human RBC elimination, involve antigenic carbohydrate moieties. Sialic-acid residues on human erythrocytes are a potential pathway for rapid monocytic clearance of red cells (Fig. 4C). Erythrocyte-specific glycophorin A (CD235a), a major sialo-glycoprotein that carries approximately 75% of the total sialic acid found in RBC membranes [151], contains terminal sialic acid residues shown to be recognized by porcine Kupffer cells [152]. Sialoadhesin, also known as CD169 or Siglec-1, is a macrophage-restricted adhesion molecule that mediates sialic-acid-dependent binding to cells [153, 154]. Petitpas *et al.* demonstrated that desialation of human erythrocytes with neuraminidase and treatment with the monoclonal antibody IF1, which blocked porcine sialoadhesin, both reduced porcine macrophage binding to human RBCs [155]. Binding was measured in “rosettes,” or clusters of ≥3 erythrocytes bound to one macrophage. Similarly, purified mouse sialoadhesin has been shown to agglutinate sheep and human erythrocytes [156].

Uniquely, due to an inactivating mutation of the CM-P-N-acetylneuraminic acid hydroxylase (CMAH) gene, humans express the Neu5Ac form of sialic acid [157]. Most other mammals, including pigs, mice, and non-human primates, dominantly express Neu5Gc [158, 159]. The deficiency of Neu5Gc in human red blood cells has been identified as a limitation for xenotransplantation models, including the use of non-human primates in preclinical xenotransplantation studies [160].

In an assay of binding between human erythrocytes and murine alveolar macrophages, Ducreux *et al.* demonstrated that pretreatment of human erythrocytes with sialidase completely abrogated rosetting, while pretreatment of murine alveolar macrophages with sialidase increased rosette formation. Their work indicated sialoadhesin masking by endogenous sialic acid on murine alveolar macrophage cell surfaces [161]. The role of murine sialoadhesin for human cell clearance, particularly RBCs, in mice is a line of research in need of further scrutiny.

## 7. IMPROVING SURVIVABILITY

Many approaches to improving the engraftment of foreign cells in immunodeficient mice have been tried to increase chimerism levels and donor cell longevity. Importantly, the limits to which mice can be humanized or otherwise made chimeric with xenogeneic cells are unknown. Efforts at genetic humanization will continue to address incompatibilities between mouse and human genes that limit the normal functioning of transplanted cells. Protocols used to construct humanized mice continue to evolve, resulting in animals engrafted with multiple cell and tissue types, which further benefits the engraftment and longevity of human cells by humanizing the overall *in vivo* environment. We will discuss some past and more recent efforts at constructing humanized mice with a focus on addressing barriers imposed by the mouse’s monocytic cells.

### 7.1. The Spleen Alone is not the Major Barrier to Xenogeneic Engraftment

The spleen has long been recognized to play an important role in the clearance of aged, damaged, or opsonized blood cells [162, 163]. Early studies performed on humans found the spleen to be dominant to the liver in the removal of compromised red cells, with longer survival times observed in individuals after splenectomy. Similarly, the survival in the circulation of modestly damaged red cells in rats was enhanced by splenectomy, but with greater damage of transfused red cells, the liver became as effective as the spleen in removing the compromised erythrocytes [164]. As the clearance of xenogeneic RBCs has been shown to differ in SCID mice depending on the species of the donor cells [73], it would not be surprising that the relative role of the spleen and liver in xenogeneic cell clearance differs based on donor species and cell type.

Splenectomy of SCID mice did not promote the survival of human RBCs in circulation [165]. However, the survival of human erythrocytes that had been parasitized with *P. falciparum* increased in NOD-SCID mice after splenectomy. In a short-term analysis of human RBC distribution in NSG mice, it was found that human cells were more likely to be sequestered in the lung and liver than in the spleen in the first minutes after transfusion [12]. Nonetheless, we note that some investigators have used splenectomy in their chimeric mouse models, although the overall justification for this procedure is unclear [101].

It is also worth noting that the spleen is often used as the site of transplantation for models aiming to generate chimeric livers, as hepatocytes and liver sinusoidal endothelial cells will travel to the liver after injection [166–174]. In such models, some cells may remain in the spleen, which may be a cause for splenectomy if these extra-hepatic donor cells are a detriment to the experiment [175].

### 7.2. Carrier Cells may offer Protection to Small Grafts

As splenectomy does not remove all monocytic cells that may interfere with xenogeneic engraftment, other methods have been devised to overwhelm or circumvent the monocytic barrier. For any transplants that involve the transport of the donor cells in the bloodstream, there is a risk that donor cells are cleared by host monocytic cells prior to engraftment. Early studies by Martino *et al.* showed that human lymphocytes were found primarily in the lungs of SCID mice after intravenous injection [176]. However, there is safety in terms of numbers as indicated by the kinetics of RBC clearance with different doses of donor cells [12]. This set of experiments showed rapid clearance of human RBCs within the first 5 minutes after transfusion into NSG mice. It was also noted that a full dose of human RBCs (1.1 x 10^9^ cells) led to chimerism levels 10 minutes after transfusion that were more than 4-fold higher than in mice transfused with half as many cells. Similar observations were made with RBC transfusions in SCID mice; it was observed that RBCs from a third species, bovine or equine, did not prolong the survival of human RBCs [73]. Thus, the mechanism of xenogeneic RBC clearance appears to have some species specificity, with C3 binding of human RBCs but not bovine or equine RBCs being observed. Overall, these findings clearly show that there is a finite capacity for xenogeneic cell clearance, which can be overwhelmed, at least briefly.

The inclusion of ‘carrier cells’ to block the clearance of a small number of donor cells has been employed as a strategy to improve the engraftment of hematopoietic stem cells [177–180]. These carrier cells usually consist of lethally irradiated cells, such as peripheral blood or whole bone marrow cells. The inclusion of carrier cells is likely to be most effective when donor cell numbers are low, although we are unaware of any formal test of this supposition. High numbers of purified donor cells or mixed populations of donor cells, such as whole bone marrow, may be less affected by pre-engraftment monocytic cell clearance. However, given the dosage effects observed with very high numbers of transfused human RBCs, the question remains of how many and what types of carrier cells would provide optimal protection for stem cell transplants.

### 7.3. Improving Engraftment by Avoiding Intravascular Administration through Direct Orthotopic or Heterotopic Transplantation

Direct injection of xenogeneic cells into the desired location can reduce the likelihood of donor cell clearance by monocytic cells, given the observations discussed above, that tissue chimerism exceeds that of blood cell chimerism in hematopoietic stem cell engrafted animals. Other cell types not associated with a natural inclination to their appropriate tissue are commonly delivered directly to their target location. For instance, placement of diverse tissue and cell types underneath the kidney capsule [181], direct injection of embryonic stem cell derivatives or hematopoietic cells into the liver parenchyma [182, 183], intracranial delivery of tumor cell lines [184], mesenchymal stromal cell (MSC) injection into the salivary glands or femoral marrow [185, 186], intra-muscular transplantation of islet cells [187], adrenal transplantation of neuroblastoma patient-derived xenografts (PDX) [188], and intra-peritoneal trans-plantation of hematopoietic cells to engraft the peritoneal cavity [176, 189] to mention just a small number of the many humanized mouse models that have been created by avoiding an intravascular route of administration.

Improved success with hematopoietic transplantation by direct intrafemoral injection rather than by the traditional intravascular route was shown by a number of investigators [190, 191]. Combined hematopoietic and MSC transplants have been performed that showed enhanced engraftment at the site of injection [192, 193]. Intrafemoral and intrahepatic injection of hematopoietic precursors developed from embryonic stem cells was also performed to address concerns that a homing defect in these cells may be the cause for the lack of engraftment [194].

### 7.4. Towards Full Replacement of Mouse Blood

An interesting short report by Tsuji *et al.* [101] has raised the possibility of the near-full replacement of mouse blood with human blood. Their work, which was performed in SCID mice, used multiple interventions to boost the levels of human red cell chimerism. Administration of autologous human serum was found to improve the survival of human erythrocytes. However, human serum was not the only treatment employed, as the mice were also splenectomized to unknown effect. Moreover, achieving the near-complete replacement of circulating mouse red cells with human red cells required multiple transfusions of human erythrocytes as well as antibody depletion of the murine red cells. Human serum was injected many times over the course of a two-week experiment, and radiation was also used as a treatment to reduce endogenous red cell production. This allowed the authors to demonstrate that mice could survive with nearly complete replacement of their circulating erythrocytes with human red cells and also that the human red cells could be infected with *Plasmodium falciparum*, thereby modeling malaria infection.

It is worth noting that although the frequency of human red cells was reported near 100%, other relevant hematological parameters, such as hematocrit and total red blood cell numbers, were not reported [101]. The effects of serum alone, heterologous human serum, the timing and dose of delivery, the effects of serum from other species, and the underlying mechanisms that account for the positive effects of the human serum have yet to be studied. The authors’ simultaneous use of various treatments also made difficult the interpretation of each individual treatment’s impact. It is reasonable to assume, given the number of treatments employed, that the health of the mice was likely adversely affected, which could impact their long-term survival and the utility of such a model. As this work was published in 1995, with no indication that it has led to further implementation of the techniques described, the results remain both aspirational and provocative until verified.

**Fig. (5).**
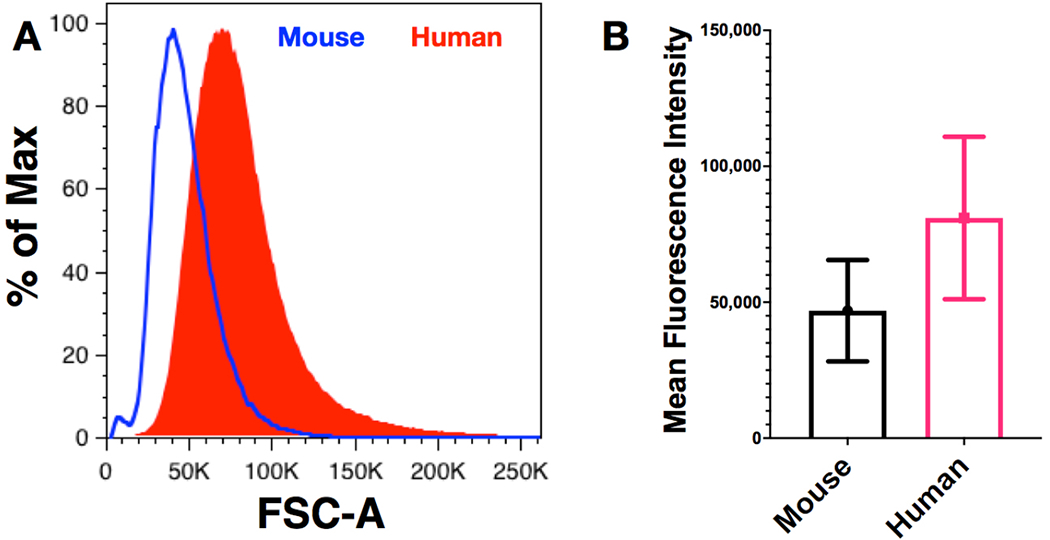
Human erythrocytes are larger than mouse erythrocytes. Flow cytometry was used to compare forward light scatter-area (FSC-A) characteristics of mouse and human RBCs. The overlay histogram data show the larger size of the human cells (A). The mean fluorescence intensity ± standard deviation of FSC-A intensity is shown as a bar plot for both cell types (B). Human and mouse red cells were mixed for analysis with the human cells identified using CD235a staining and mouse cells identified by enhanced green fluorescence protein expression derived from a transgenic marker expressed in all tissues. Mouse RBCs were purified from whole blood prior to mixing with human cells. Data are from [12]. (*A higher resolution / colour version of this figure is available in the electronic copy of the article*).

The prospect that human erythrocytes may fully substitute for mouse erythrocytes raises an issue, often debated, regarding the physical limitations that may limit the circulation of human cells in mice. Human and mouse erythrocytes have key structural and physiological differences that have been suggested to play a role in human RBC rejection from murine circulation. For example, human erythrocytes are 7.3 microns in diameter on average, with a mean corpuscular volume of 95 cubic microns. Mouse erythrocytes average 5.8 microns in diameter, with a mean corpuscular volume of 41 cubic microns [195]. Indeed, this size discrepancy is readily observable by flow cytometry as differences in forward light scatter (Fig. 5). This size difference is in concert with the difference in size between human and murine capillaries (the typical human capillary diameter is 8 microns, while murine capillaries average 4 microns in diameter) [196], which could possibly play a role in accelerating human cell clearance. Additionally, certain rheological properties differ between human and murine erythrocytes; mouse RBCs are more deformable [197], and mouse RBCs demonstrate lower aggregability [198]. Rheological determinants, such as aggregability, encourage the axial flow of erythrocytes in blood vessels, which, in turn, increases leukocyte margination and encourages leukocyte adhesion [198–200]. The potential relationship between erythrocyte rheology and human cell clearance in humanized mouse models is currently not well understood.

### 7.5. Combinational Approaches to Maximizing Engraftment

A mouse model described by Rongvaux *et al.* [70] represents a notable achievement in mouse humanization. On a *Rag2^null^ Ilrg^null^* background, Rongvaux *et al.* constructed the MISTRG mouse with homozygous knock-ins of human cytokines M-CSF, IL3, thrombopoietin, and G-CSF, as well as a human SIRPα bacterial artificial chromosome transgene. Knocking in cytokines, as opposed to inserting viral promoter-driven transgenes or plasmids, was intended to circumvent previously observed issues associated with cytokine overexpression in transgenic strains [201]. This model conferred potential competitive advantages to transplanted human cells and was shown to result in a ten-fold improvement of human myeloid cell engraftment compared to humanized NSG mice. Importantly, the significance of self--recognition *via* SIRPα and CD47 cross-reactivity was evaluated through the construction of MITRG, a variant of MISTRG without the human SIRPα transgene. Although MITRG mice engrafted with human HSC contained lower numbers of human NK cells compared to MISTRG mice, overall myeloid engraftment was similar between the two strains, indicating that advantages conferred by the cytokine knock-ins, including potentially more supportive developmental microenvironments, eclipsed the importance of SIRPα-driven phagocyte recognition. However, the authors noted this model’s tendency to become anemic within a short time frame, especially following radiation and higher levels of human cell engraftment. The cause of the observed anemia was proposed to be the potential consumption of mouse erythrocytes by human phagocytes, as well as low levels of both murine and human erythropoiesis.

Further work on the MISTRG mouse by Song *et al.* [202] addressed the insufficient human erythrocyte reconstitution previously observed in humanized MISTRG mice and other humanized mouse models. The authors humanized the MISTRG mouse liver using a *Fah* deletion and intrasplenic hepatocyte injection construct, where mice with damaged livers more easily accommodated human hepatocyte engraftment [203]. The liver produced the majority of innate immune defense proteins, including complement C3, and housed large populations of tissue-resident macrophages (Kupffer cells) as well as monocyte-derived macrophages. Humanization of the mouse liver was subsequently shown to decrease murine complement C3 opsonization of human RBCs, reduce the number of mouse macrophages in the liver, and increase the recruitment of human monocyte-derived macrophages to the liver. Survival of transfused human RBCs in circulation was markedly improved in human hepatocyte-engrafted mice, but the majority still ended up in the liver, as previously observed [12]. Additionally, human erythropoiesis in the bone marrow was observed to be improved, with some erythroblastic islands composed of human central macrophages and human erythrocytes. However, the terminal enucleation rate, as defined by the percentage of enucleated human RBC in the bone marrow, remained static between liver-humanized and non-liver-humanized MISTRG mice, indicating that liver humanization does not improve the end stages of human erythropoiesis. Analysis of human RBCs in murine peripheral blood for HSC-engrafted humanized-hepatocyte mice showed human RBCs occupying around 1% of peripheral blood. Although a notable improvement was observed compared to erythrocyte reconstitution in other strains [71], many barriers still need to be overcome in order to create a humanized mouse model that can be said to possess human blood.

## 8. FUTURE DEVELOPMENTS

Further improvements in the humanization of mice will very likely involve utilizing multiple approaches, both genetic and transplant-based, to address current deficiencies. In terms of further humanization of the hematopoietic and immune systems, addressing the issue of species-specificity of many different growth factors is needed. However, this approach alone is often insufficient. For instance, defects in the maturation of human B-cells were thought to be the result of a lack of activity of murine B-cell activation factor (BAFF) in human cells. However, humanizing BAFF did not resolve the problem [204]. As observed with MISTRG mice, human erythropoiesis is limited even in the presence of humanized cytokines and human cells. We discuss some possibilities to further improve xenogeneic cell engraftment based on targeting mouse monocytic cells.

### 8.1. Generating Immunodeficient Mice that Enable Targeted Depletion of Monocytic Cells

Transient and reversible depletion of mouse macrophages in adult mice would likely be useful to further improve engraftment and to better define the role of macrophages in graft rejection. As discussed, the depletion of macrophages in mice is currently most commonly carried out through the use of clodronate liposomes [113]. However, clodronate is known to be toxic and even fatal to mice in large, repeated doses [205], which are necessary to achieve adequate macrophage ablation levels for enhanced human engraftment. Moreover, clodronate liposomes do not distinguish between mouse and human cells. Murine myeloid cells also have proliferative advantages over human cells and thus will rapidly reconstitute the depleted monocytic cells to the disadvantage of human cells [206–208]. Consequently, if mouse cells can recover faster, there is no way to boost the engraftment of human cells or cells of another species with clodronate liposomes. An alternative method is to utilize transgenic strains, such as MaFIA, which express a Fas-based suicide gene and have greater macrophage-ablation specificity [115]. Importantly, mouse-specific ablation of monocytic cells could be performed after xenografting. Our laboratory is investigating a line of immunodeficient mice containing the MaFIA construct; early experiments suggest that human blood cell survival is enhanced after macrophage depletion to the degree expected from previous studies using clodronate liposome treatment (the work of Hui *el al.* is in progress).

### 8.2. Supporting Human Myelopoiesis to Enhance Overall Human Engraftment

Beyond the depletion of host monocytic cells, replacing these cells with donor cells would be ideal. Donor-derived monocytic cells support engraftment not just by eliminating xenogeneic phagocytosis but also by providing human growth signals to donor cells. This has been shown in NSG-3GS mice that have elevated levels of human myeloid cells in response to the presence of human growth factors, which leads indirectly to improved T- and NK-cell engraftment [67, 71, 209]. Another interesting aspect to be considered is the supportive role macrophages may play as part of the hematopoietic stem cell niche, which is disrupted by their depletion [210]. Substitution of host macrophages with donor macrophages in the hematopoietic niche could, therefore, possibly improve our modeling of *in vivo* human hematopoiesis.

The lack of activity of some murine growth factors on human cells will most likely need to be addressed, as human myelopoiesis is not fully supported in mice due to the lack of cross-reactivity of GM-CSF and M-CSF [211]. Human CD14^+^ monocytes do develop in mice and can be found in the bone marrow [60, 212], blood [213], spleen [71], and liver [214]. Human monocyte maturation, however, was found to be reduced in mice [215]. However, the levels and functional maturity of human monocytes are increased when human cytokines are provided [70, 185, 213, 216–229].

## CONCLUSION

Innate immune barriers remain one of the hurdles to generating humanized mice, with cells of the monocyte lineage being the primary barrier. Developing better methods to at least temporally deplete mouse monocytic cells while supporting the engraftment of human cells may offer an approach to further increase human chimerism. However, such efforts may uncover that monocytic cells may not be the only remaining barriers to xenogeneic cell survival in mice. Other factors are likely at play, and it is anticipated that continued improvements in the humanization of mice through optimized cell and tissue transplantation, the creation of genetically improved mouse lines that express human growth factors, and the targeted inhibition or depletion of host monocytic cells will lead to greater human chimerism. These efforts will be driven by the fact that further improvements in humanized mouse models will foster many areas of biomedical research.

## Figures and Tables

**Table 1.** Available strains of immunodeficient mice for use as hosts for human cell and tissue transplantation.

Common Name	Full Name	Phenotype	Source	References
NSG / NOD-*Scid*-gamma	NOD.Cg-*Prkdc*^*scid*^ *Il2rg*^*tm1Wjl*^/SzJ	Absent B, T, and NK cells. Defective dendritic and macrophage cells. Lacks hemolytic complement activity.	Jackson Laboratory (Bar Harbor, ME, USA)	[59, 60]
NSG-3GS / NSGS / NSG-SGM3	NOD.Cg-*Prkdc*^*scid*^ *Il2rg*^*tm1Wjl*^ Tg(CMV-IL3,CSF2,KITLG)1Eav/MloySzJ	Express human interleukin-3 (IL-3), granulocyte-macrophage colony-stimulating factor (GM-CSF), and stem cell factor (SCF) on an NSG background.	Jackson Laboratory (Bar Harbor, ME, USA)	[209, 213, 218, 219]
hSCF-Tg-NSG / hKL-NSG	NOD.Cg-*Prkdc*^*scid*^ *Il2rg*^*tm1Wjl*^ Tg(PGK1-KITLG* 220)441Daw/SzJ	Express human membrane-bound stem cell factor (hSCF or KITLG) on an NSG background.	Jackson Laboratory (Bar Harbor, ME, USA)	[68, 213, 220]
NBSGW / NSGW	NOD.Cg-*Kit^W-41J^ Tyr* + *Prkdc^scid^ Il2rgt^m1Wjl^*/ThomJ	NSG background. Support engraftment of human hematopoietic cells without irradiation.	Jackson Laboratory (Bar Harbor, ME, USA)	[10, 60, 221]
NCG	NOD-*Prkdc^em26Cd52^ Il2rg^em26Cd22^*/NjuCrl	Lack functional B, T, and NK cells. Reduced macrophage and dendritic cell function.	Charles River (Wilmington, MA, USA)	[222]
B-NDG	NOD.CB17-*Prkdc*^*scid*^ *Il2rg*^*tm1*^/Bcgen	Absent B, T, and NK cells. Defective dendritic and macrophage cells. Lacks hemolytic complement activity.	Biocytogen (Wakefield, MA)	-
NOG	NOD.Cg-*Prkdc^scid^ Il2rgt^m1Sug^*/ShiJic	Absent B, T, and NK cells. Reduced macrophage and dendritic cell function. Lack hemolytic compliment activity.	CIEA (Kawasaki, Japan) Taconic Biosciences (Germantown, NY, USA)	[223]
DKO	C57BL/6NTac.Cg-*Rag2^tm1Fwa^ Il2rgt*^*m1Wjl*^	Absent B, T, and NK cells.	Taconic Biosciences (Germantown, NY, USA) Jackson Laboratory (Bar Harbor, ME, USA)	[64]
BRG / Rag2^−/−^ γc*−/−*	C;129S4-*Rag2^tm1.1Flv^ Il2rg*^*tm1.1Flv*^/J	Absent B, T, and NK cells.	Jackson Laboratory (Bar Harbor, ME, USA)	[66]
hSIRPa-DKO	C;129S4-*Rag2^tm1.1Flv^ Il2rg^tm1.1Flv^* Tg(SIRPA)1Flv/J	Express human signal-regulatory protein alpha (SIRPα) in addition to BRG defects.	Jackson Laboratory (Bar Harbor, MN, USA)	[69]
MISTRG	C;129S4-*Rag2*^*tm1.1Flv*^ *Csfl*^*tm1(CSF1)Flv*^ *Csf2/Il3*^*tm1.1 (CSF2,IL3) Flv*^ *Thpo*^*tm1.1 (TPO)Flv*^ *Il2rg*^*tm1.1 Flv*^ Tg(SIRPA)1Flv/J	Express human M-CSF, IL3, TPO, and SIRPα on Rag2^−/−^ γc^−/−^ background	Jackson Laboratory (Bar Harbor, MN, USA)	[70]
NRG / NOD rag gamma	NOD.Cg-*Rag1*^*tm1Mom*^ *Il2rg*^*tm1Wjl*^/SzJ	Absent B, T, and NK cells. Defective dendritic and macrophage cells. High irradiation tolerance.	Jackson Laboratory (Bar Harbor, MN, USA)	[224–226]
BRGSF	BALB/c *Rag2−/−Il2rg−/− Sirpa*NOD*Flk2−/−*	Express NOD Sirpa, defects in hematopoietic progentor development. Absent B, T, and NK cells.	genOway (Lyon, France)	[227–229]

## LIST OF ABBREVIATIONS

BAFF: B-cell Activation Factor
CIEA: Central Institute for Experimental Animals
CMAH: CMP-N-Acetylneuraminic Acid Hydroxylase
Crry: CR1-Related y
DAF: Decay Accelerating Factor
DC: Dendritic Cell
FL: Flk-2/flt3 Ligand
GM-CSF: Granulocyte-Macrophage Colony-Stimulating Factor
HSC: Hematopoietic Stem Cell
IFN: Interferon
IFNGR2: IFN-Gamma Receptor 2
IL: Interleukin
LPS: Lipopolysaccharide
M-CSF: Macrophage Colony-Stimulating Factor
MaFIA: Macrophage Fas-induced Apoptosis
MCP: Membrane Cofactor of Proteolysis
MHC: Major Histocompatibility Complex
MSC: Mesenchymal Stromal Cell
NK: Natural Killer
NOD: Non-Obese Diabetic
NSG: NOD-*Scid*-Gamma
PBMCs: Peripheral Blood Mononuclear Cells
PDX: Patient-Derived Xenografts
RAG: Recombination-Activating Genes
RBC: Red Blood Cell
SCF/KL: Stem Cell Factor/Kit Ligand
SCID: Severe Combined Immunodeficiency
SIRPα: Signal Regulatory Protein Alpha
STAT1: Signal Transducer/Activator of Transcription 1
TNFα: Tumor Necrosis Factor Alpha
TPO: Thrombopoietin
